# Biventricular repair of pulmonary atresia with intact ventricular septum and severely hypoplastic right ventricle: a case report of a minimum intervention surgical approach

**DOI:** 10.1186/s13019-016-0486-z

**Published:** 2016-07-04

**Authors:** Hiroaki Hata, Naokata Sumitomo, Mamoru Ayusawa, Motomi Shiono

**Affiliations:** Department of Cardiac Surgery, Nihon University School of Medicine, 30-1 Oyaguchikamimachi, Itabashi-ku, Tokyo, 173-8610 Japan; Department of Pediatric Cardiology, Saitama Medical University International Medical Center, 1397-1 Yamane, Hidaka, 350-1298 Japan; Department of Pediatrics, Nihon University School of Medicine, Tokyo, Japan

**Keywords:** Pulmonary atresia with intact ventricular septum, Central shunt, Reconstruction of the right ventricular outflow tract, Polytetrafluoroethylene bulging valved conduit, Artificial patent foramen ovale

## Abstract

**Background:**

In patients who have pulmonary atresia with an intact ventricular septum and severe right ventricular hypoplasia, biventricular repair is considered to be impossible and multiple interventions are generally required for definitive repair.

**Case presentation:**

An initial palliative procedure was performed in a 1-month-old boy to promote right ventricular development by pulmonary valvectomy without disrupting the annulus, and appropriate oxygenation was achieved with a central funnel shunt. The retained annulus caused functional stenosis and prevented unfavorable right ventricular dilatation due to regurgitation. Thirteen years later, without any other intervention, reconstruction of the right ventricular outflow tract was successfully performed for definitive biventricular repair by using a new expanded polytetrafluoroethylene bulging valved conduit with extended longevity.

**Conclusions:**

The successful outcome in this case suggests that our minimal palliation strategy could be one option for management of these patients.

## Background

If biventricular repair is possible with a single second surgical procedure, it is the most desirable strategy for patients who have pulmonary atresia with an intact ventricular septum (PAIVS) and severe hypoplasia of the right ventricle (RV). While transcatheter valvotomy is also useful, the majority of survivors require further intervention [1]. When biventricular repair is considered, pulmonary valvotomy and/or patch plasty is performed initially to promote the growth of the RV, and a systemic-pulmonary shunt is added if pulmonary blood flow is inadequate [2, 3]. The definitive procedure is usually performed by age four [2], although revision of the right ventricular outflow tract (RVOT) is often necessary as the patient grows. We report a rare case of definitive repair at the age of 13 years without any other intervention after an initial palliative procedure at 1 month. The good outcome in our patient suggests that this could be one of the surgical strategies to consider for PAIVS.

## Case presentation

**Fig. 1:**
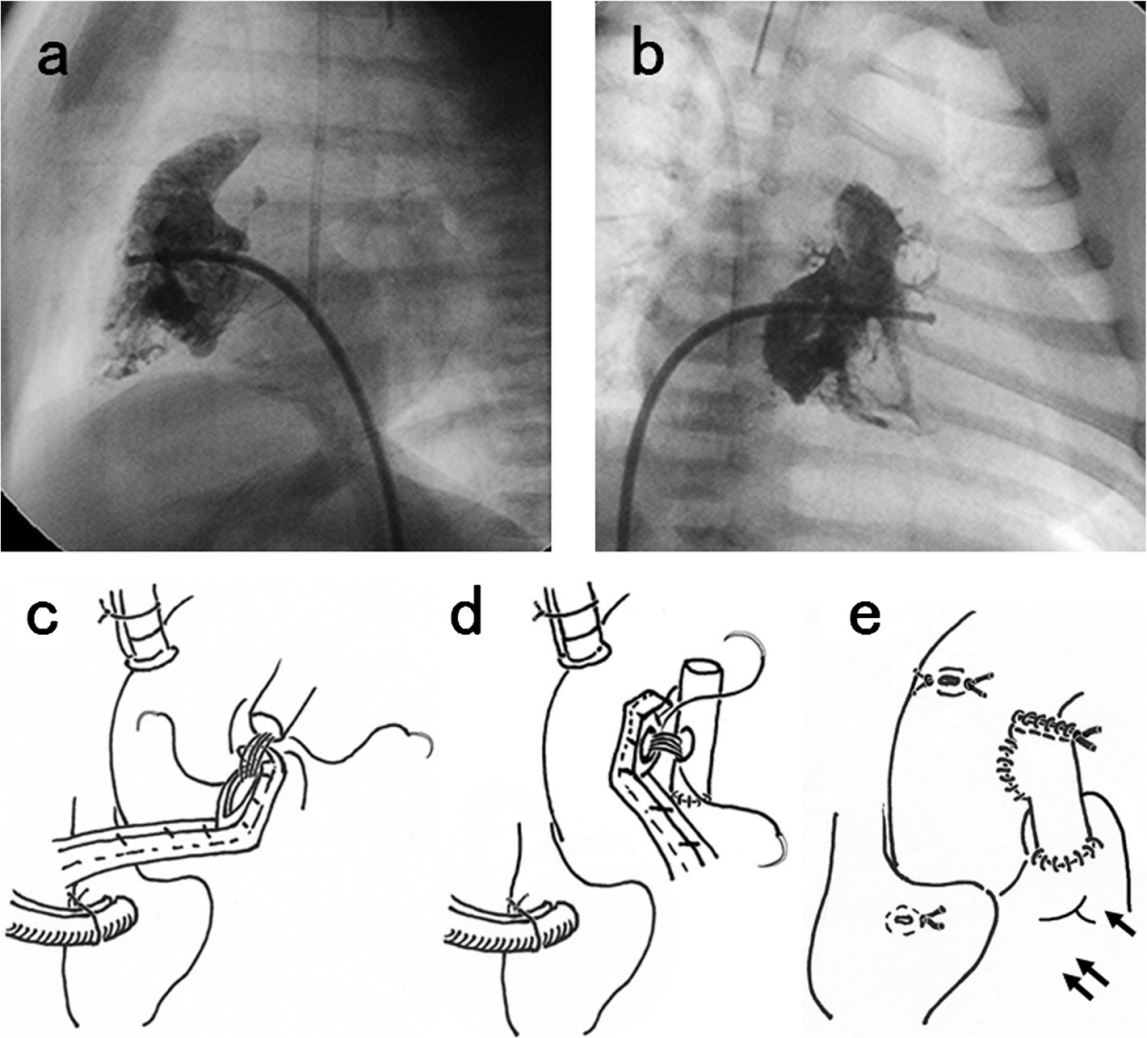
Preoperative right ventriculography showed that the end-diastolic volume was 22 % of normal and the Z-score of the tricuspid valve and pulmonary valve was −5.4 and −3.8, respectively. (**a** anterior, b lateral). **c** The distal end of a 3.5 mm polytetrafluoroethylene graft was attached to the main pulmonary artery (←) by end-to-side anastomosis. **d** The graft was attached tangentially to the left side of the ascending aorta by side-to-side anastomosis. **e** The proximal stump of the graft was closed, creating a funnel-shaped graft (funnel shunt) to avoid kinking. (⇇): Right ventricular outflow tract (RVOT)

In a 1-month-old boy weighing 3.1 kg, both PAIVS and ductus-dependent pulmonary circulation were diagnosed. A right ventriculogram revealed the infundibulum and showed minor sinusoidal communications. The end-diastolic volume of the tripartite RV was only 22 % of normal, while the diameter of the tricuspid valve and pulmonary valve was 8.4 and 5.3 mm, respectively. The Z-score (standard deviation unit) [4] of the tricuspid valve and pulmonary valve was −5.4 and −3.8, respectively. (Fig. 1a-b). Intervention for pulmonary atresia by catheter perforation of the valve was unsuccessful because of a shortage of strength of the catheter wire. In March 1997, at the age of 1 month, the patient underwent beating heart surgery using a heart-lung machine. The patent ductus arteriosus was divided and transpulmonary complete pulmonary valvectomy was done without disrupting the annulus. Patch plasty of the RVOT was not performed. To secure adequate pulmonary blood flow, a central shunt was created with a 3.5 mm polytetrafluoroethylene graft. In order to maintain long-term patency, end-to-side anastomosis was performed between the distal end of the graft and the main pulmonary artery (Fig. 1c), followed by tangential side-to-side anastomosis with the left side of the ascending aorta (Fig. 1d). Then the proximal stump of the graft was closed (Fig. 1e) to create a funnel-shaped shunt and avoid kinking [5].

Various formulae are used for quantitative assessment of right ventricular morphology. It has been reported that the Z-score of the tricuspid valve is strongly correlated with right ventricular cavity size [6]. In patients with normal coronary circulation but a very small tricuspid valve (Z-score of less than −4), concomitant transannular patching and systemic-pulmonary artery shunting is indicated as the initial procedure despite the high risk [6]. In our patient, the initial tricuspid valve Z-score was −5.4. The index of right ventricular development (RVDI) [2, 7] and the right ventricular index (RVI) [3] are employed during initial palliative procedures. If RVDI is <0.35 or the ventricular cavity is small (RVI <11), valvotomy with a concomitant Blalock-Taussig shunt is required [2, 3, 7]. In our patient the RVDI and RVI when palliative repair was performed were 0.33 and 10.4, respectively. After the initial procedure, peripheral oxygenation was well maintained and growth delay was not noted. Therefore, the patient was followed up carefully without further intervention.

**Fig. 2:**
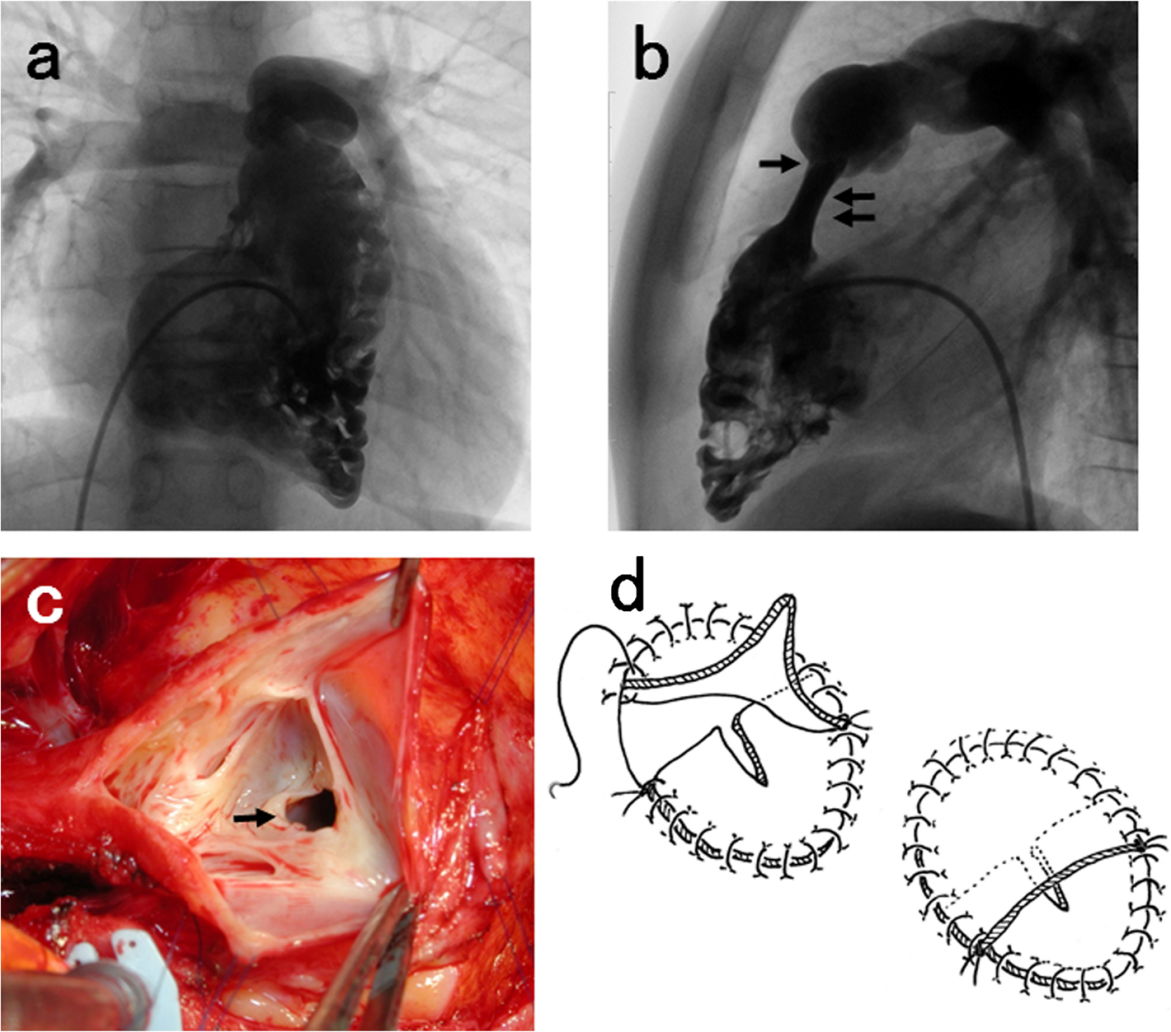
After 13 years, right ventriculography revealed an obviously tripartite ventricle with stenosis of the pulmonary annulus (➝) and RVOT (⇇) (**a** anterior, **b** lateral). **c** Intraoperative view of the stenosed pulmonary annulus (➝). **d** *Left*: The atrial septal defect was partly closed from each side with bilayer patches of autologous pericardium that overlapped at the center of the defect. An 8 mm slit was cut in the patch on the left atrial side with a scalpel as a vent to reduce right heart overload. *Right*: The patch on the right atrial side was sutured to overlap the left atrial side patch by about 5 mm

Cardiac catheterization was performed at the age of 12 years because of a gradual increase in cyanosis. The body weight, pulmonary/systemic blood flow ratio, pulmonary artery pressure, right ventricular pressure, left ventricular pressure and arterial oxygen saturation were 43 kg, 1.3, 18/5 mmHg, 85/7 mmHg, 138/10 mmHg, and 92 %, respectively. Echocardiography revealed that the RVOT gradient was 64 mmHg. An atrial septal defect and proximal stenosis of the right pulmonary artery were noted. The central shunt was patent, with minimum flow, though pulmonary blood flow was mostly supplied from the RV (Fig. 2a). Desaturation became worse if the central shunt was occluded with a balloon. RVOT stenosis was caused by the retained pulmonary annulus and the long constricting elastic muscular tissue of the infundibulum (Fig. 2b), which was impossible to dilate by catheter intervention. Mild pulmonary regurgitation and trivial tricuspid regurgitation were detected. The RV-tricuspid valve index (RV-TVI) is used for assessment of definitive surgery and biventricular repair is considered to be possible when this index is >0.4 [2, 7]. The right ventricular end-diastolic volume was 46.1 % of normal, RV-TVI was 0.75, and the tricuspid valve Z-score was 2.6. Although the RV was relatively small, it had a tripartite morphology and it was judged that biventricular repair would be possible. In October 2010, the central shunt was excised under cardiopulmonary bypass. After cardiac arrest, the stenosed right pulmonary artery was enlarged with autologous pericardium and reconstruction of the RVOT was done (Fig. 2c) with an in situ expanded polytetrafluoroethylene valved conduit (22 mm in diameter), which is large enough for most Japanese adults. This conduit was developed by Miyazaki and associates [8]. It has bulging sinuses to generate vortex flow similar to that created by the sinus of Valsalva, which promotes closure of the native semilunar valves and reduces wear and stress, thus increasing valve longevity [9]. The atrial septal defect was partly closed from each side with bilayer patches of autologous pericardium that overlapped at the center of the defect. An 8 mm slit was cut in the patch on the left atrial side with a scalpel as a vent to reduce right heart overload by acting like a patent foramen ovale, after which the patch on the right atrial side was sutured to overlap that on the left atrial side by about 5 mm (Fig. 2d).

**Fig. 3:**
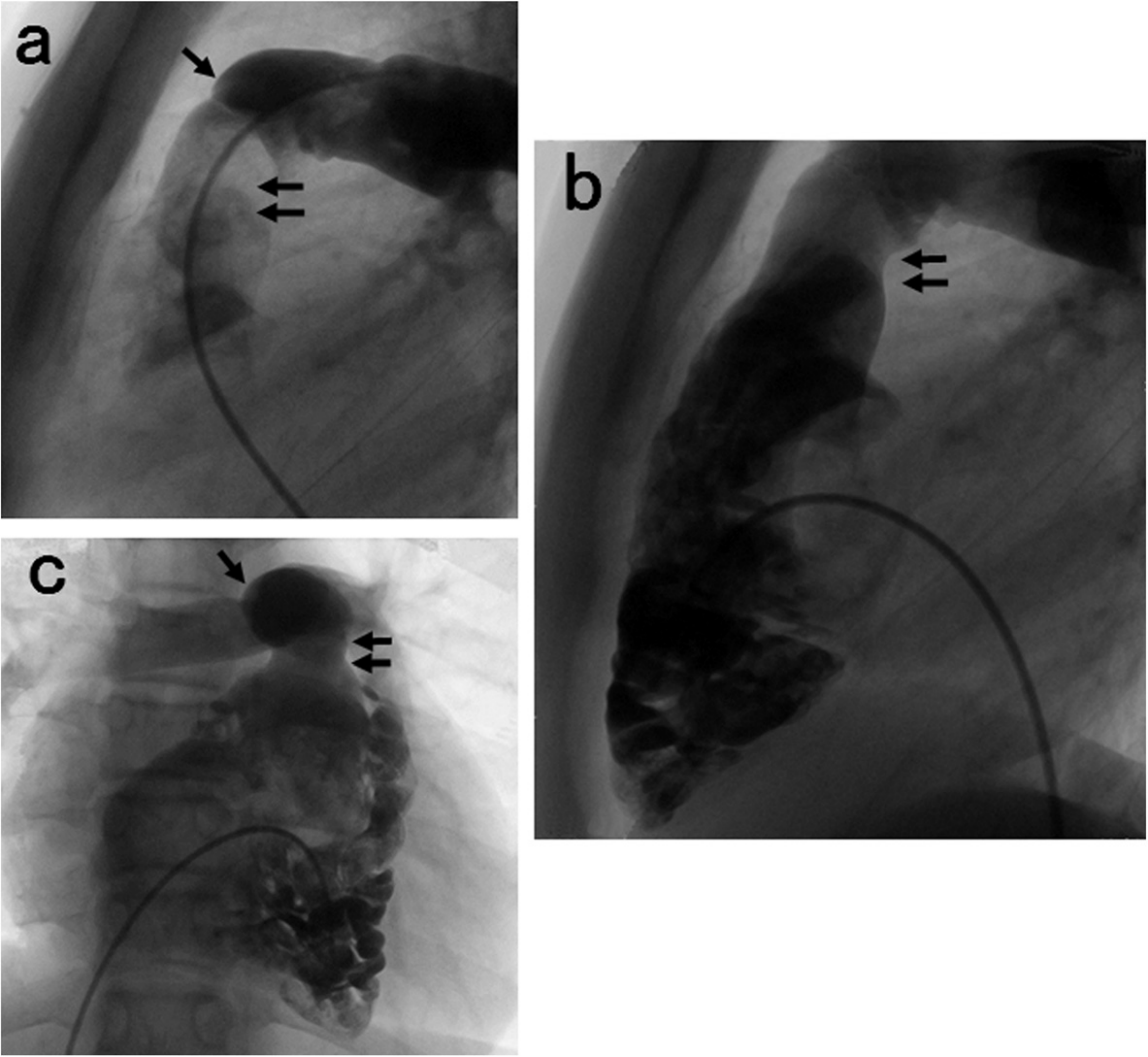
**a b** Postoperative lateral view of the main pulmonary artery (➝). **c** Postoperative anterior right ventriculogram. The right ventricular outflow tract (⇇) has almost normal morphology with minor catheter-induced pulmonary regurgitation

Cyanosis resolved by 3 days postoperatively, suggesting that the vent (artificial patent foramen ovale) was effective. One year after definitive repair, both RVOT morphology and conduit function were satisfactory (Fig. 3a-c). Five years after repair, the tricuspid valve diameter and Z-score were 41 mm and 1.1, respectively, while arterial oxygen saturation was 98 %.

### Discussion

In patients with PAIVS, a two-stage surgical approach is generally employed to optimize growth of the RV and tricuspid valve [2, 6, 7]. Catheter pulmonary valvotomy would probably have left more severe residual pulmonary stenosis than surgical valvectomy, which would have decreased flow through the RV and thus diminished the growth potential of the RV. In our patient, growth of the hypoplastic RV was promoted by securing RV-pulmonary artery continuity via complete pulmonary valvectomy without disrupting the annulus through addition of a central funnel shunt. The retained annulus had the same effect as functional valvular stenosis while maintaining the required amount of pulmonary flow and unfavorable right ventricular dilatation due to regurgitation was avoided. After this procedure, stable hemodynamics were maintained until the patient was 13 years old. If a definitive procedure is performed during infancy, revision of the RVOT will eventually be required. RVOT reconstruction with pulmonary valve replacement is one of the solutions for right ventricular volume overload, but its timing is controversial because of the surgical risk and limited life of prosthetic valves. Severe chronic pulmonary regurgitation and associated dilation and dysfunction of the RV have been the main focus of several recent investigations in patients with PAIVS [10]. The incidence of severe pulmonary regurgitation after repair with a transannular patch was reported to be about 30 % at the age of 22 years [11]. Although nontransannular patches are reported to show better durability without disrupting the pulmonary annulus, revision of the RVOT is still required [12]. In PAIVS patients undergoing late pulmonary valve replacement after palliative surgery, management of significant tricuspid regurgitation is commonly required [10]. While a causative relationship between pulmonary regurgitation tricuspid regurgitation has not been established, definitive repair with a competent pulmonary valve is crucial to minimize RV dilatation that may precipitate tricuspid regurgitation. In our patient, definitive biventricular repair could be performed at 13 years old by using a competent expanded polytetrafluoroethylene bulging valved conduit without earlier intervention. Atrial venting (creation of an artificial patent foramen ovale) was helpful during the acute postoperative period. Five years after the procedure, the patient is studying at university and can play football.

## Conclusion

This report describes how we performed definitive bivetricular repair in a boy at 13 years without any other intervention after an initial palliative procedure at 1 month. The successful outcome in the present patient suggests that our strategy with minimum palliative procedure could be one option for the management of PAIVS.

## Abbreviations

PAIVS, pulmonary atresia and an intact ventricular septum; RV, the right ventricle; RVOT, the right ventricular outflow tract; RVDI, the index of right ventricular development; RVI, the right ventricular index; RV-TVI, the RV-tricuspid valve index.
